# Psychometric validation of the school health assessment tool for primary schools in Southeast Nigeria

**DOI:** 10.1186/s12889-025-24279-7

**Published:** 2025-09-02

**Authors:** Danilo Garcia, JohnBosco Chika Chukwuorji, Maryam Kazemitabar, Tochi E. Iwuagwu, Chinyere Chibuko, Ricardo Sanmartin, Claudia Chiarolanza, İsmail Seçer, Kevin M. Cloninger

**Affiliations:** 1 Department of Social Studies, University of Stavanger, Stavanger, Norway; 2 Department of Behavioral Sciences and Learning, Linköping University, Linköping, Sweden; 3 Department of Social Studies, Promotion of Health and Innovation for Well-Being (PHI-WELL), University of Stavanger, Stavanger, Norway; 4 Lab for Biopsychosocial Personality Research (BPS-PR), International Network for Well- Being, Stavanger, Norway; 5 Promotion of Health and Innovation (PHI) Lab, International Network for Well-Being, Linköping, Sweden; 6 Centre for Ethics, Law and Mental Health (CELAM), University of Gothenburg, Gothenburg, Sweden; 7 Department of Psychology, University of Gothenburg, Gothenburg, Sweden; 8 Department of Psychology, University of Nigeria, Nsukka, Enugu state Nigeria; 9 Yale School of Medicine, Yale University, New Haven, Connecticut, USA; 10 Department of Human Kinetics and Health Education, University of Nigeria, Nsukka, Enugu state Nigeria; 11 Guidance & Counseling Unit at the Post-primary Schools Management Board, Enugu, Nigeria; 12 Department of Developmental Psychology and Didactics, Faculty of Education, University of Alicante, Alicante, Spain; 13 Department of Dynamic and Clinical Psychology, Sapienza - University of Rome, Rome, Italy; 14 Department of Psychological Counseling and Guidance, Atatürk University, Erzurum, Turkey; 15 College of Health Sciences, University of Missouri, Columbia, MO USA; 16 Anthropedia Foundation, St. Louis, MO USA

**Keywords:** Educational settings, Nigeria, Psychometric validation, Public health, School health assessment, School policy, SHAT-PS

## Abstract

**Background:**

School health programs are critical for promoting the physical, psychological, and social well-being of students and staff, particularly in low- and middle-income countries. Effective evaluation of these programs requires comprehensive and context-sensitive tools. The School Health Assessment Tool for Primary Schools (SHAT-PS), originally developed and validated in Iran, offers a biopsychosocial framework for evaluating key dimensions of school health. Designed for use in low- and middle-resource settings and emphasizing both staff and student well-being, the SHAT-PS provides a promising foundation for addressing gaps in school health assessment. However, although this tool has demonstrated strong psychometric properties in its original context, its applicability in other cultural and educational settings remains underexplored.

**Aim:**

This study aimed to validate the SHAT-PS in Nigerian primary schools by examining its factor structure, internal consistency, and contextual relevance.

**Method:**

A total of 706 primary school teachers and school staff from the Nsukka education zone in southeast Nigeria completed the original 76 items of the SHAT-PS. Although the tool was originally developed in Persian, an English version, produced by the original developers through a standard forward and back translation process, was available and used in this study. As English is the official language of instruction in Nigeria, no additional translation was required. Instead, adaptation efforts focused on ensuring contextual and conceptual relevance of the items for the Nigerian educational setting, including expert review and evaluation by Nigerian educational psychologists and school administrators. We employed a cross-sectional design and conducted an Exploratory factor analysis (EFA) on half of the sample and a confirmatory factor analysis (CFA) on the remaining half. Internal consistency was assessed using Cronbach’s alpha (α).

**Results:**

EFA and CFA supported a six-factor structure (52 items): (1) School Health Policies, (2) Health Education and Psychological Services, (3) Health Services, Nutrition, and Staff Health, (4) School Hygiene, (5) School Area and Comfort, and (6) Physical Environment. The tool demonstrated good internal consistency (α = 0.66-0.89) and strong construct validity. Variations in responses highlighted its sensitivity to local discrepancies in infrastructure, services, and staff well-being.

**Conclusion:**

The SHAT-PS was successfully validated for use in Nigerian primary schools. Its robust psychometric performance and contextual alignment with national school health priorities make it a valuable tool for monitor school health and inform targeted interventions. Further validation in other regions and among broader stakeholder groups is recommended to enhance its generalizability and support nationwide implementation.

## Introduction

According to the World Health Organization (WHO), a health-promoting school is one that continuously strengthens its capacity to provide a healthy learning, working, and living environment [1, 2]. Informed school health programs are therefore critical for promoting the physical, psychological, and social well-being of students and school staff. Effective school health programs have, for example, been linked to improved academic performance, reduced absenteeism, and healthier lifestyle choices among students [3–12]. In support of such efforts, the United Nantions’ Sustainable Development Goals emphasize inclusive, equitable, and quality education as a foundation for lifelong well-being, particularly for children in vulnerable situations [13]. Globally, school health promotion models such as the WHO’s Global School Health Initiative and the U.S. Centers for Disease Control and Prevention’s Whole School, Whole Community, Whole Child model advocate for comprehensive approaches that incorporate physical education, health services, nutrition, psychological support, and family-community engagement [14, 15]. Despite these advances, the ability to assess these multidimensional domains of school health remains limited in many low- and middle-income countries, especially in Africa. The lack of contextually adapted, psychometrically sound assessment tools hampers efforts to monitor school health systems and implement data-driven improvements [16].

One promising instrument is the School Health Assessment Tool for Primary Schools (SHAT-PS), developed in Iran through a rigorous exploratory sequential mixed-method design [14]. The SHAT-PS assesses eight key domains of school health: (1) School Health Policies, (2) Community Connections, (3) Health Education, (4) Physical Activity, (5) Health Services, (6) Nutrition, (7) Psychological Services, and (8) Physical Environment [14]. It has demonstrated strong psychometric properties in its original (Persian) validation in Iran, including high internal consistency, sound construct validity, and high test-retest reliability [14]. Compared to other tools (e.g., School Health Program Evaluation Scale, Healthy School Indicator Tool, and School Health Evaluation Instrument), the SHAT-PS adopts a more integrative biopsychosocial framework. It places greater emphasis on psychological services and staff well-being and was explicitly designed with low- and middle-income countries in mind [14, 17]. Importantly, the SHAT-PS was developed specifically for primary school settings. This focus reflects both practical and theoretical considerations: primary schools are typically more accessible across populations, serve the largest share of students in many low- and middle-income countries, and play a foundational role in shaping lifelong health behaviors. Moreover, in primary settings, teachers often take on broader responsibilities, including informal health education and psychosocial support, which makes them key informants in assessing school health environments. These contextual and policy-driven features informed the tool’s design and its emphasis on teacher-reported indicators [14].

However, applying the SHAT-PS in other low- and middle-income countries requires, as a first step, validation of its basic psychometric properties, including factor structure and internal consistency. Given the limited availability of validated instruments that align with the five-component framework of the Nigeria’s National School Health Policy, namely, (1) health services, (2) skill-based health education, (3) a healthful school environment, (4) school feeding services, and (5) school–community relationships [18]; we found the SHAT-PS particularly suitable for validation in the Nigerian context.

### School health in Nigeria

The Federal Republic of Nigeria, a West African country comprising 36 states and the Federal Capital Territory, operates a 6-3-3 educational system structure, six years of primary education, followed by three years each of junior and senior secondary education. Basic education, which includes both primary and junior secondary schools, is officially free and compulsory in public schools. English is the official language of instruction. Oversight is provided centrally by the Universal Basic Education Commission and implemented through State Universal Basic Education Boards and Local Educational Authorities. Students are assessed through continuous evaluations and term-end exams, culminating in the Common Entrance Examination, which determines entry into secondary education and confers the First School Leaving Certificate. Improving school health within this system is not only a public health priority but also a critical strategy for enhancing educational quality and equity. Indeed, with more than 71% of the 47 million basic education students enrolled at the primary levels [19, 20] primary schools in Nigeria are key platforms for implementing health interventions. Effective school health programming could enhance student learning outcomes, foster safe and inclusive environments, and reduce the burden on Nigeria’s overstretched health system through early prevention.

To address these opportunities and challenges, Nigeria’s National School Health Policy, as previously outlined, proposed a five-component model for advancing school health: (1) school health services, (2) skill-based health education, (3) healthful school environment, (4) school feeding services, and (5) school, home, and community relationships [18]. Despite, and perhaps partly due to, its comprehensive design, the policy has faced significant implementation challenges. Evidence suggests that many schools often lack routine health screenings, pre-enrollment medical evaluations, and consistent health education [21, 22]. Other widespread issues include inadequate hygiene infrastructure and a shortage of trained school health personnel [18]. These limitations directly undermine students’ physical, psychological, and social development and negatively affect educational performance and equity. For instance, only 63% of children regularly attend primary school, and fewer than 84% transitioning to junior secondary education after completion [23]. These implementation gaps reflect not only limited resources, but also the lack of standardized tools to monitor policy compliance and guide improvement.

A psychometrically sound instrument like the SHAT-PS could help address these shortcomings. By offering structured, quantifiable insights into key areas of school health, it can support evidence-based planning, identify differences across schools and regions, and facilitate policy accountability among education stakeholders. When validated for local use, the SHAT-PS offers significant potential for large-scale applications to evaluate implementation fidelity and guide targeted interventions effectively.

### The present study

In this study, we evaluated the SHAT-PS for use in Nigeria, examining its psychometric properties and contextual suitability for assessing school health among Nigerian primary school teachers and staff. Instead of relying on the 47 items retained in the original Iranian factor structure, we included all 76 items initially developed by Kazemitabar and colleagues [14]. This decision, aligned with their recommendation to use the complete item pool in cross-cultural adaptation studies, allowed us to assess whether a similar or contextually unique factor structure in Nigeria would emerge. Although the tool was originally developed in Persian, an English version, produced by the original developers through a standard forward and back translation process [14] was available and used in this study. As English is the official language of instruction in Nigeria, no additional translation was required. Instead, adaptation efforts focused on ensuring contextual and conceptual relevance of the items for the Nigerian educational setting, including expert review and evaluation by Nigerian educational psychologists and school administrators. Notably, one Nigerian co-author of the present study also contributed to the original SHAT-PS validation, and two additional Nigerian co-authors are experienced school and educational psychologists, thereby supporting cultural alignment and content validity.

The SHAT-PS was selected for two primary reasons, in addition to its biopsychosocial theoretical foundation and strong psychometric performance in Iran. First, its core domains closely reflect the five components of Nigeria’s National School Health Policy [18] which emphasizes school health services, a healthful school environment, skill-based health education, and collaboration with families and communities. Second, the SHAT-PS was specifically designed for low- and middle-income countries and is intended to be completed by teachers, who are recognized in Nigerian policy as key stakeholders in school-based health promotion [24].

## Methods

### Ethical statement

 This study was conducted in accordance with standard ethical principles and protocols, ensuring participants anonymity and confidentiality of data. Schools’ teachers and staff participation was voluntary. The study protocol was reviewed and approved by the Research and Ethics Committee, Department of Psychology, University of Nigeria, Nsukka (approval number: D.PSY.UNN/REC/2021-4-000029-2). Written informed consent to participate was obtained from all participants prior to their inclusion in the study, in accordance with the ethical guidelines outlined in the Declaration of Helsinki.

### Participants and procedure

 A total of 706 teachers (87.9% females, 12.1% males) were recruited from primary schools in the Nsukka educational zone, which is one of the six education zones in the Enugu state, southeast Nigeria. The SHAT-PS, 76 items, was administered in February 2024 during teachers’ regular term meetings. Participants responded to the survey using a four-point Likert scale ranging from "*not any*" to "*very much*".

### Adaptation process of the SHAT-PS

 The SHAT-PS, originally developed and validated in Persian in Iran, was adapted for use in Nigeria using guidelines informed by the COSMIN framework for cross-cultural adaptation of outcome measures [25]. An English version had already been created by the original developers for use in international collaboration and publication. This initial translation followed conventional guidelines for instrument adaptation, including forward and back translation, expert review, and item equivalence checks [14]. Since English is the official language of instruction in Nigerian primary schools, and all participants were fluent in English, our adaptation in the present study built on this existing English version, focusing on ensuring semantic, contextual, and conceptual relevance within the Nigerian primary school setting, rather than performing additional linguistic translation procedures. Specifically, we followed these key adaptation steps:

1. Three Nigerian educational and school psychologists independently reviewed all 76 original SHAT-PS items to assess conceptual clarity, cultural appropriateness, and relevance to the Nigerian educational system. Items were compared to Nigerian school health policies and curriculum content.
2. Each item was evaluated for contextual suitability using a content validity checklist focused on school infrastructure, service norms, and policy alignment. Items deemed culturally inappropriate, ambiguous, or irrelevant were flagged for discussion.
3. A consensus meeting was held among Nigerian co-authors, psychologists, and school administrators to reconcile discrepancies and make minor wording adjustments where necessary.

### Statistical analysis

We performed data cleaning to ensure the absence of duplicate or incomplete responses. The dataset was split randomly into two halves: one half was used for the exploratory factor analysis (EFA) to identify the factor structure, and the other for the confirmatory factor analysis (CFA) to validate the identified structure. The overall statistical approach was structured to (1) examine item distribution and internal consistency, (2) identify the underlying factor structure through EFA, (3) confirm this structure through CFA, and (4) assess the reliability of the resulting scale. All statistical analyses were conducted using SPSS v24 for descriptive statistics and EFA, and R v4.4.2 with RStudio for CFA.

### Descriptive statistics

 We calculated frequencies and percentages for all categorical sociodemographic variables (e.g., gender, marital status, employment type) and means and standard deviations for continuous variables (e.g., age, years of employment). These results are summarized in Table 1.

**Table 1:** Sociodemographic characteristics and descriptive statistics of the study sample

Variable		*N*	Percentage
Gender	Female	580	87.9%
	Male	80	12.1%
Age - Mean and SD (±)	40.25 ± 9.85	506	71.7%
Marital status	Living with another	32	5.0%
	Married	468	72.9%
	Divorced	6	0.9%
	Separated	11	1.7%
	Single	62	9.7%
	Widowed	63	9.8%
Employment status	Permanent	596	93.7%
	Temporal/Parent Teacher Association teacher	40	6.3%
Yearly income	Less than 360,000 NGN	301	51.2%
	360,000–500,000 NGN	186	31.6%
	500,000 to 1 million NGN	62	10.5%
	Above 1 million NGN	39	6.6%
Position	Principal	11	1.7%
	Head Teacher	98	15.3%
	Class Teacher	507	79.0%
	Administrative staff	16	2.5%
Education level	Secondary school graduate	17	2.7%
	TC-II	6	0.9%
	NCE/OND	312	49.1%
	Bachelor’s degree/HND	266	41.9%
	Graduate degree (e.g., Masters, Doctorate)	34	5.4%
Employment Years - Mean and SD (±)	14.12 ± 11.47	589	83.4%

Percentages account only for available data (i.e., some variables have missing data)

### Assessment of normality

Prior to factor analyses, we assessed the univariate distribution of all 76 SHAT-PS items using skewness and kurtosis statistics. Items with values outside ±2 were flagged for review but retained to preserve content coverage and comparability with the original version.

### Item-total correlations

 Pearson’s item-total correlations were computed to evaluate the relationship between each item and the overall scale score. Items with weak correlations (*r *<.20) were noted and considered during factor refinement.

### Exploratory and confirmatory factor analyses

The first half of the dataset was subjected to EFA using maximum likelihood estimation with an oblimin rotation to allow for correlated factors. Factors with eigenvalues >1.0 were retained, and solutions explaining ≥82% of the cumulative variance were considered. Items with low factor loadings (< 0.40) or cross-loadings (< 0.40) on multiple factors were reviewed for potential removal to enhance model fit and interpretability​.

 Using the second half of the data, we conducted CFA, for the model structure from the EFA, with the weighted least squares mean, and variance adjusted (WLSMV) estimator, suitable for ordinal and non-normal data. Fit indices such as comparative fit index (CFI), Tucker-Lewis index (TLI), root mean square error of approximation (RMSEA), and standardized root mean residual (SRMR) were evaluated to assess model fit​​.

### Reliability analysis

 Internal consistency of each factor and the total scale was assessed using Cronbach’s alpha (α). A threshold of ≥ 0.60 was considered acceptable for research purposes.

## Results

### Descriptive statistics and normality Estimation

The descriptive statistics of the sample are presented in Table 1. Normality assumptions were evaluated through skewness and kurtosis statistics. While most items fell within acceptable limits of ± 2, some items exhibited slight deviations, indicating potential non-normality. Given the robust nature of the subsequent analyses, these deviations were not considered severe enough to warrant item removal.

### Exploratory factor analysis

**Fig. 1:**
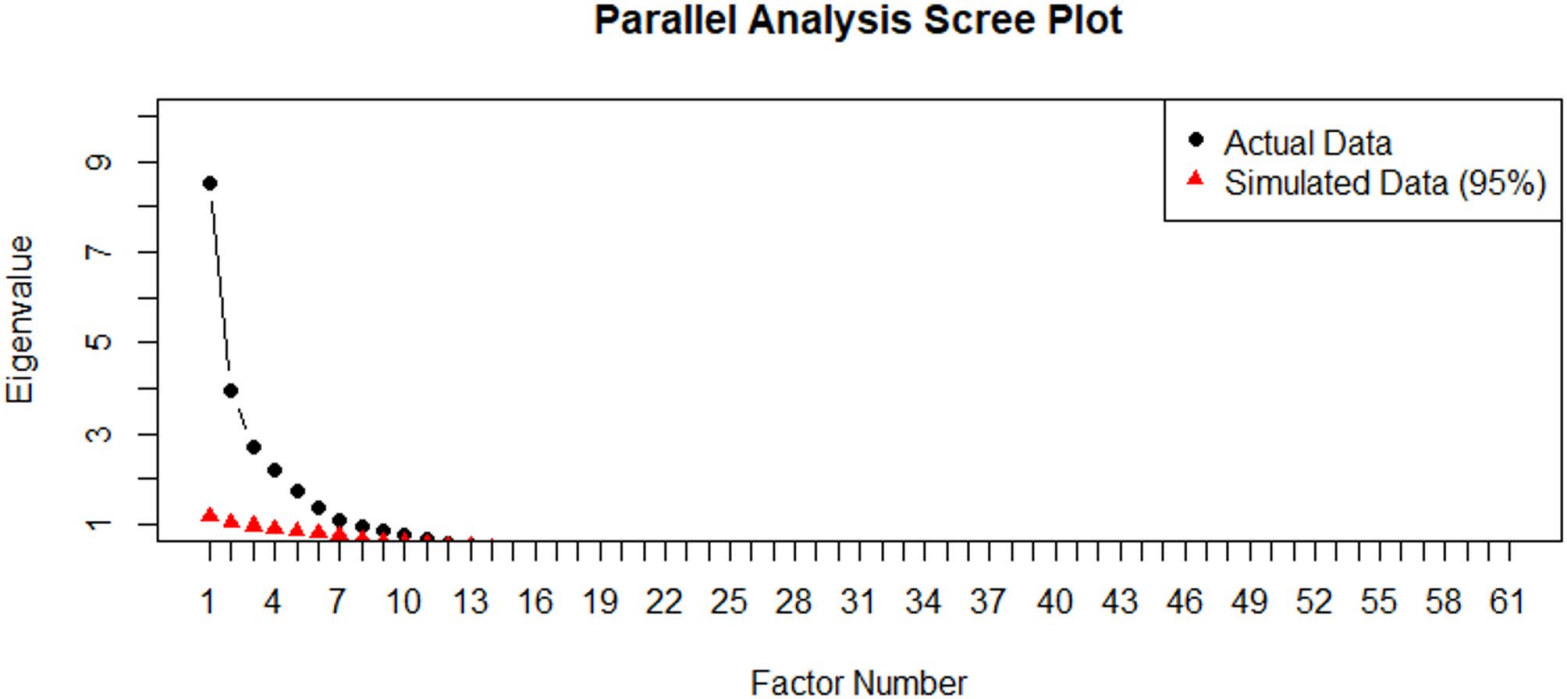
Parallel analysis scree plot comparing observed eigenvalues (black dots) with simulated eigenvalues (red triangles) Note. The inflection point after the seventh factor supports a seven-factor solution; however, based on conceptual and structural requirement of at least three items per factor, a six-factor model was retained

Using the first half of the data, we conducted an EFA using maximum likelihood extraction and oblimin rotation​. For interpretability, we examined factor solutions ranging from two to eight factors. Parallel analysis, which compares observed eigenvalues to those generated from random data, supported the retention of seven factors based on the criterion that actual eigenvalues exceed simulated ones up to the seventh factor. This was consistent with Kaiser’s criterion (eigenvalues > 1.00) and supported by the inflection point observed in the parallel analysis scree plot (Fig. 1). However. the seven-factor model included one factor (Factor 7) composed of only two items, which does not meet the conventional minimum of three items per factor required for structural stability. Therefore, these two items, items 37 and 61, were reassigned to more conceptually appropriate factors (Factor 2 and Factor 6 respectively). The resulting six-factor model retained strong conceptual coherence and improved structural integrity.

The six retained factors were labeled as follows: “School Health Policies”, “Health Education and Psychological Services”, “Health Services, Nutrition and School Staff’s Health”, “School Hygiene”, “School Area and Comfort”, and “Physical Environment”. Table 2 presents the standardized loadings for the 61 retained items, all of which loaded ≥ 0.40 on their respective factors (loadings ranging from 0.40 to 0.97). Inter-factor correlations ranged from 0.01 to 0.35, indicating weak to moderate associations without multicollinearity. Item-total correlations were all significant at *p* <.01, except for item 61 (*p* >.05).

**Table 2:** Item-level standardized factor loadings from the seven-factor EFA, including all 61 retained items

Item #	Factor 1	Factor 2	Factor 3	Factor 4	Factor 5	Factor 6	Factor 7	Item-total correlation
1	0.42	−0.01	0.10	0.08	0.03	0.01	0.17	0.26
2	0.03	0.41	−0.02	0.08	0.07	−0.01	0.10	0.29
3	0.50	−0.08	0.08	0.03	−0.10	−0.14	−0.15	0.14
4	0.52	−0.12	0.11	−0.06	0.33	−0.02	0.21	0.25
5	0.57	−0.10	0.10	−0.01	0.07	0.02	0.21	0.26
6	0.59	0.05	0.08	0.02	0.13	−0.07	0.13	0.33
7	0.49	0.24	−0.12	−0.07	0.02	0.07	0.14	0.32
8	0.49	0.19	−0.20	0.08	−0.15	0.15	−0.03	0.29
9	0.61	0.02	−0.16	0.24	−0.10	0.01	−0.17	0.30
10	0.02	0.45	−0.04	−0.19	0.01	0.02	−0.08	0.17
11	−0.06	0.54	−0.21	0.01	−0.06	0.16	0.03	0.22
12	0.48	0.12	0.00	−0.11	0.07	0.16	−0.07	0.32
13	0.66	−0.12	0.13	0.01	−0.03	0.15	0.10	0.30
14	0.44	0.14	−0.08	0.11	−0.10	0.16	−0.03	0.31
15	0.40	0.38	−0.06	0.08	0.07	−0.01	−0.09	0.43
16	0.05	0.46	0.18	0.22	−0.03	−0.19	−0.08	0.40
17	0.09	0.46	0.09	−0.02	−0.04	0.13	−0.09	0.41
18	0.14	0.47	0.35	−0.26	−0.01	0.03	0.01	0.40
19	0.09	0.58	0.27	−0.13	0.04	−0.04	−0.09	0.42
20	0.13	0.47	−0.01	−0.2	0.04	0.17	0.03	0.27
21	−0.02	0.74	−0.07	0.10	0.07	−0.05	−0.08	0.43
22	−0.05	0.66	−0.13	0.07	0.10	0.04	0.07	0.37
23	0.30	0.28	0.40	−0.10	0.10	−0.05	−0.06	0.42
24	0.33	−0.12	0.53	0.12	−0.09	−0.06	−0.07	0.23
25	0.33	0.11	0.41	0.02	−0.17	0.09	−0.06	0.36
26	0.06	0.44	0.14	−0.02	0.00	0.08	0.06	0.38
27	0.19	0.05	0.48	0.07	−0.07	0.11	0.13	0.32
28	−0.05	0.35	0.45	0.11	−0.27	0.03	0.09	0.38
29	−0.05	0.11	0.52	0.10	−0.08	0.23	0.19	0.42
30	0.13	−0.06	0.53	0.16	−0.13	0.10	0.11	0.36
31	0.00	−0.08	0.47	0.25	−0.21	0.01	0.10	0.25
32	0.05	−0.02	0.62	0.16	−0.21	0.14	0.02	0.32
33	−0.02	0.03	0.62	0.16	−0.21	0.24	0.02	0.42
34	0.02	0.07	0.52	0.13	−0.07	0.10	−0.16	0.34
35	−0.02	0.42	−0.14	0.09	0.02	0.15	0.11	0.25
36	−0.17	0.47	−0.13	0.05	0.09	0.18	0.26	0.22
37	0.35	−0.01	0.14	0.02	−0.02	0.22	−0.44	0.27
38	−0.09	0.44	0.07	0.12	−0.04	0.22	0.07	0.37
39	0.41	0.20	−0.15	0.10	0.07	0.12	−0.05	0.34
40	−0.09	0.44	−0.11	0.31	0.18	−0.05	0.25	0.32
41	−0.08	0.07	0.07	0.80	0.00	0.01	−0.07	0.41
42	0.06	0.01	0.01	0.89	0.05	0.02	0.03	0.45
43	0.05	−0.02	0.13	0.61	0.01	0.12	−0.17	0.41
44	0.21	0.02	0.09	0.42	0.09	0.29	−0.08	0.49
45	−0.04	0.11	−0.18	0.29	0.12	0.46	0.18	0.36
46	−0.01	0.02	0.00	0.22	0.16	0.45	0.22	0.35
47	0.07	−0.06	0.12	0.22	0.00	0.57	−0.07	0.41
48	0.04	0.01	0.12	−0.01	−0.01	0.88	−0.23	0.50
49	0.06	0.08	−0.08	−0.10	0.26	0.61	0.08	0.36
50	0.11	−0.13	−0.14	0.10	0.75	0.15	0.05	0.23
51	−0.04	0.05	0.02	0.02	0.84	0.01	−0.01	0.27
52	0.03	0.17	0.02	−0.04	0.77	−0.04	−0.10	0.30
53	−0.16	0.08	0.20	0.14	0.48	0.10	−0.15	0.36
54	−0.16	0.11	0.29	0.13	0.44	0.15	0.00	0.41
55	−0.11	−0.02	0.60	−0.01	0.32	−0.01	0.07	0.34
56	0.00	−0.09	0.79	0.06	0.12	0.08	−0.11	0.41
57	0.12	−0.05	0.61	0.24	0.11	0.03	−0.10	0.42
58	0.09	−0.01	0.58	0.00	0.19	0.10	−0.23	0.37
59	0.10	−0.04	0.47	0.02	0.35	−0.01	0.20	0.29
60	0.13	0.00	0.47	0.03	0.17	0.02	0.05	0.35
61	0.09	0.00	0.04	−0.05	−0.04	−0.11	0.97	−0.05
% Variance	34.42%	14.38%	9.88%	7.76%	6.40%	4.94%	4.55%	

### Confirmatory factor analysis

**Fig. 2:**
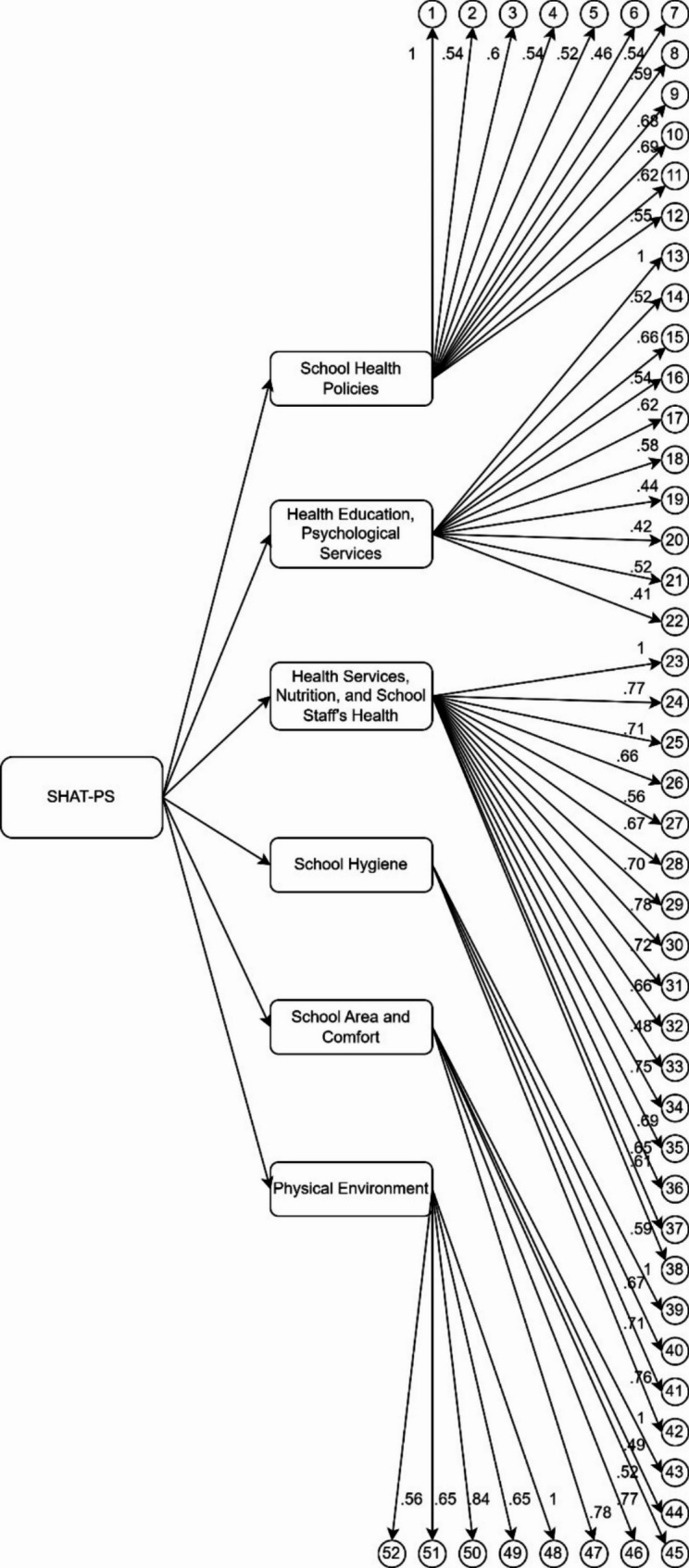
CFA path diagram of the six-factor model with standardized factor loadings for 52 items

The second half of the data was used to validate the EFA six-factor structure through CFA using the WLSMV estimator to account for the ordinal nature of the responses. The fit indices suggested an acceptable model fit: Chi-square (*χ*²) = 1901.73, df = 1242, *p* <.001, CFI = 0.90, TLI = 0.89, RMSEA = 0.04 (90% CI: 0.04–0.05), SRMR = 0.09. Of the 61 items retained from EFA, nine with factor loadings below 0.40 were excluded, resulting in a final scale of 52 items. The standardized factor loadings in the final model ranged from 0.41 to 0.84, confirming the structural robustness of the six-factor model (see Fig. 2; Table 3 for the details). The covariances between latent factors ranged from 0.17 to 0.48, suggesting moderate to strong relationships among the school health dimensions. These CFA results support the multi-dimensionality and construct validity of the SHAT-PS for use in Nigerian primary schools.

**Table 3:** Final Nigerian version of the school health assessment tool for primary schools (SHAT-PS)

Factor	Item #	Statements
School Health Policies	1	Health educational posters, stands and boards have been installed in classrooms, hallways and halls
	2	There is a distinctive health organization at the school (such as health care providers, health pioneers, and health promoters)
	3	The school’s educational facilities are sufficient
	4	The school’s amenities are sufficient
	5	The school has regular programs for out-of-school recreational and educational activities for students
	6	Families work with the school to improve students’ health
	7	Supportive and charitable organizations work with the school to promote school health
	8	Media and TV provide educational programs to promote health at the school
	9	The school organizes health-related educational workshops, conventions, and programs for students
	10	The school organizes health-related educational workshops, conventions, and programs for parents
	11	The school organizes health-related educational workshops, conventions, and programs for school staff
	12	First aid training is provided to students
Health Education, Psychological Services	13	The school has specific rules for the rights and duties of individuals
	14	Health-related routine behaviors are taught to students
	15	Adequate hours during the week are devoted to health education
	16	There is sufficient course or syllabus for health education
	17	There is a skilled physical education teacher at school
	18	At least one sport is taught professionally at school, such as volleyball, basketball, handball, football, etc
	19	Adequate hours of the week are devoted to sport at school for students
	20	Teachers share students’ health/educational issues with their parents
	21	How to communicate and interact healthily and effectively is taught to students
	22	Students enjoy attending the school
Health Services, Nutrition, and School Staff’s Health	23	Students’ health status is assessed and registered in their health records
	24	There is a skilled nurse practitioner/physician at the school
	25	The school nurse(s) provide emergency health care services to students and school staff
	26	The school has an equipped place to provide health services
	27	The school meals are prepared according to hygienic principles
	28	The school offers healthy and nutritious foods
	29	Healthy and safe drinking water is available
	30	The school has buffet or restaurant with enough space to sit
	31	The buffet’s hygiene is monitored
	32	Foods and snacks are served according to students’ tastes
	33	The school has a well-equipped library
	34	The school has a well-equipped laboratory
	35	Psychological services are provided for school staff
	36	Sport facilities are provided for school staff
	37	The school staff’s job satisfaction is met
	38	Teachers receive adequate salaries
School Hygiene	39	Restrooms are clean
	40	The number of restrooms is sufficient
	41	Drinking fountains or water coolers are clean
	42	The number of drinking fountains is sufficient
School Area and Comfort	43	Classroom desks and chairs are standard and comfortable
	44	The area of the school is proportional to the number of students
	45	The area of the classrooms is proportional to the number of students
	46	The school has an appropriate manner for sanitary waste disposal
	47	The school’s physical environment is happy (colors, decorations, layouts etc.)
Physical Environment	48	The number of ventilators in different parts of the school is sufficient
	49	Classroom lighting is sufficient
	50	Heating system in classrooms is appropriate and sufficient
	51	Cooling system in classrooms is appropriate and sufficient
	52	The school is regularly inspected for the safety of buildings, windows and equipment

Originally published in Kazemitabar, M., Garcia, D., Chukwuorji, J. C., SanMartín, R., Lucchese, F., Khoshnood, K., & Cloninger, K. M. (2021). Development and Primary Validation of the School Health Assessment Tool for Primary Schools (SHAT-PS). *PeerJ*, 9:e12610. https://10.7717/peerj.12610. For any use, research or commercial, please contact D. Garcia, danilo.garcia@icloud.com, and M. Kazemitabar, maryam.kazemitabar@yale.edu

### Internal consistency

Across the factors, Cronbach’s alpha coefficients ranged from 0.66 to 0.89, indicating acceptable to strong internal consistency (Table 4). The “School Health Policies” factor, comprising 12 items, achieved an alpha of 0.80, while the “Health Services, Nutrition, and School Staff’s Health” factor exhibited the highest alpha (α = 0.89) across 16 items. The “Physical Environment” factor, composed of five items addressing aspects such as ventilation, lighting, temperature regulation, and structural safety, showed the lowest reliability (α = 0.66). Although within the acceptable range for research, this moderate reliability suggests the need for further refinement for these items.

**Table 4:** Internal consistency (Cronbach’s alpha) of the six factors in the six-factor CFA solution

Factor	Number of items	Cronbach’s alpha
School Health Policies	12	0.80
Health Education, Psychological Services	10	0.75
Health Services, Nutrition, and School Staff’s Health	16	0.89
School Hygiene	4	0.68
School Area and Comfort	5	0.78
Physical Environment	5	0.66

## Discussion

The validation of the SHAT-PS in Nigeria highlights both the strengths and challenges associated with implementing school health assessment tools in low- and middle-income countries. This study showed that the SHAT-PS, originally developed in Iran, demonstrates acceptable psychometric properties when applied in Nigerian primary schools, with generally good internal consistency. The CFA supported the six-factor model found by the EFA, the model fit indices suggested an acceptable, but not excellent fit. This is particularly relevant given the large sample size, which can inflate the sensitivity of certain model fit tests, such as the chi-square statistic. Nonetheless, further refinement of the factor structure may be warranted to improve its precision.

Notably, the six identified factors (i.e., School Health Policies, Health Education and Psychological Services, Health Services, Nutrition, School Hygiene, and Physical Environment), closely align with the core domains outlined in Nigeria’s National School Health Policy, which includes (1) health services, (2) skill-based health education, (3) a healthful school environment, (4) school feeding services, and (5) school-community relationships [18]. These findings underscore the SHAT-PS’ potential to measure, and potentially inform, school health practices; thereby supporting the development of tailored, context-sensitive interventions in Nigerian educational settings. Indeed, while the policy offers a valuable framework, its broad guidelines can be difficult to translate into actionable practices, especially in a context where many schools lack routine health screenings, adequate health education resources, or sufficient medical support [21, 22]. The SHAT-PS, when further validated, can be useful to addresses this gap by operationalizing policy principles into concrete, measurable indicators that reflect how school health components are implemented on the ground. For example, instead of vaguely promoting a “healthful school environment,” the tool assesses specific elements such as classroom ventilation, lighting, and safety inspections. It also disaggregates broader domains like health education and psychological services into observable practices, making these constructs more accessible for evaluation. Importantly, the factor structure emerging from the Nigerian context offers additional value by integrating psychological services within the broader domain of health education, reflecting local needs and interpretations. By providing reliable scores across domains, the SHAT-PS might facilitate routine monitoring, pin-point implementation gaps, and eventually inform targeted interventions. In doing so, it might help to bridge the persistent gap between national policy goals and everyday school realities through evidence-based guidance.

A remarkable distinction between the Iranian and Nigerian validations of the SHAT-PS lies in the treatment of staff health. In the Iranian sample, items related to staff health were excluded from the final model due to poor factor loadings, primarily attributed to the minimal response variance—most participants reported uniformly low scores. This uniformity likely reflected the systematic neglect of staff health in Iranian primary schools, rather than irrelevance of the construct [14]. In contrast, the Nigerian validation retained this dimension, capturing meaningful variation in responses. This suggests that staff health, while still a concern, is more variable; and thus, psychometrically measurable in Nigerian primary schools. Its successful inclusion as a distinct and reliable factor demonstrates the value of cross-cultural validation, where local contextual differences shape which dimensions emerge as statistically robust. Importantly, this finding validates the decision to retain the full original pool of SHAT-PS items, rather than rely on the Iranian factor structure. It also serves as a reminder that low factor loadings in one context may stem from uniformly shared experiences, not from conceptual inadequacy. The staff health items, as all other initial items, were grounded in qualitative interviews of 65 stakeholders (including five professors of educational psychology, five principals, 18 teachers, 17 school staff, and 20 students), and therefore reflect a critical concern that may manifest differently across settings [14]. Their exclusion in the Iranian model likely reflects more uniformly low prioritization or provision of staff health services, which restricted response variance and precluded factor information. These findings highlight how local policy uptake and school-level differences can influence the psychometric structure of assessment tools. They also illustrate how tools like the SHAT-PS can be meaningfully adapted to reflect local priorities and differences, ultimately enhancing their relevance and utility in diverse educational settings.

Furthermore, as in the original Iranian validation, findings from the Nigerian context emphasize the central importance of psychological and counseling services in schools [14]. In the present study, the “Health Education and Psychological Services” factor reflected the critical need for both mental health support and educational programming, which are essential for improving student well-being and addressing behavioral and emotional issues. This is particularly relevant in settings where mental health resources and infrastructure are scarce. Nigerian schools often face challenges such as anxiety, depression, and other mental health concerns among students, often worsened by a shortage of trained counselors and school-based psychological services [4]. The inclusion of this factor in the Nigerian SHAT-PS highlights its utility in identifying service gaps and informing strategic resource allocation. Addressing these needs is essential, not only for improving individual student outcomes, but also for cultivating a healthier, more supportive school environment.

The strong internal consistency observed for the “Health Services, Nutrition, and Staff Health” (α = 0.89) factor, along with three other factors with α ranging from 0.75 to 0.89, support the reliability of the SHAT-PS across these key domains. However, the “Physical Environment” factor demonstrated lower reliability (α = 0.66), indicating weaker internal coherence among its five items. This may reflect two issues. Firstly, the broad thematic scope of the factor (e.g., ventilation, lighting, temperature control, and building safety) may combine domains that are not conceptually or functionally equivalent. Secondly, substantial infrastructural variability across schools in the Nsukka region may influence how consistently these items are interpreted and endorsed. For instance, ventilation and cooling might be more salient in some regions, while lighting and safety inspections might vary dramatically between urban and rural settings. Such divergence in relevance and implementation may reduce the statistical association between items, even if each is meaningful on its own. To improve psychometric performance, future iterations of the SHAT-PS should consider refining the item pool by examining item-total correlations, re-evaluating the dimensional structure of the factor, and gathering qualitative input from stakeholders to explore whether additional or revised items could better capture infrastructural realities of Nigerian schools.

### Limitations and strengths

Despite its strengths, this study has several limitations that offer directions for future research. First the validation of the SHAT-PS was limited to primary schools. While this focus aligns with the tool’s original design, developed specifically to capture health-related dimensions most salient in primary school settings, it restricts generalizability. Health challenges and institutional structures differ significantly across developmental stages; therefore, future research should examine the tool’s applicability and validity in secondary schools and other educational settings by adapting the SHAT-PS content to reflect age-appropriate health priorities, engaging relevant stakeholders, and conducting separate psychometric evaluations to ensure structural and contextual relevance. Moreover, although the adaptation process involved expert review by Nigerian psychologists and school administrators, providing robust contextual insight and alignment with national school health policies, the instrument was not formally pretested with typical end-users (e.g., through cognitive interviews or pilot testing). Future validation efforts should include respondent pretesting to further strengthen item clarity and ensure interpretations are consistent with frontline school staff perspectives.

Second, the data were collected exclusively from teachers and school staff. While these stakeholders are well-positioned to report on institutional practices and infrastructure, the absence of perspectives from students, parents, and community members limits the comprehensiveness of the findings. Including these voices in future research would help ensure a more holistic understanding of school dynamics and support the development of more inclusive and effective interventions. Additionally, the sample was drawn solely from Nsukka, located in the southeast region of Nigeria. While this region offers practical and contextual relevance, Nigeria is marked by deep cultural, linguistic, and infrastructural diversity across its six geopolitical zones. As such, the regional specificity of the sample may affect the generalizability and transferability of our findings to the diversity of geographic, cultural, or socio-economic conditions across the country. Future validation efforts should replicate the study across multiple geopolitical zones to examine whether the psychometric properties of the SHAT-PS remain stable or require further contextual adaptation in different regions within Nigeria.

Third, as mentioned earlier, the lower internal consistency observed for the Physical Environment factor (α = 0.66) suggests limited cohesiveness among items. This may be due to both conceptual heterogeneity (i.e., combining ventilation, lighting, climate control, and structural safety) and regional disparities in infrastructure across schools within Nsukka, which can affect how items are perceived and reported. Such variability can reduce the reliability of responses. Future research should examine item-level functioning, explore potential sub-dimensions within the domain, and involve local stakeholders to refine and expand the item set to better reflect diverse school environments. Additionally, longitudinal studies are needed to assess the SHAT-PS’s capacity to track changes in school health practices over time and to evaluate its potential impact on policy, resource allocation, and student well-being. Nonetheless, while other measures exist, such as the Scale for Health-Promoting Schools developed in Korea [25] and tools aligned with the WHO’s Health-Promoting Schools framework [26]many of these tools were developed for higher-income contexts and may lack cultural alignment with low- and middle-income countries such as Nigeria [14, 17]. The SHAT-PS stands out for its unique biopsychosocial foundation, its design for low- and middle-income countries context, and its inclusion of overlooked dimensions such as staff well-being and psychological services. Originally developed through an exploratory sequential mixed-method approach, the SHAT-PS incorporates the insight of stakeholders, enhancing its relevance and adaptability. Its strong psychometric performance in the original [14] and in the present study further supports its value as a potential diagnostic and planning tool in school health promotion.

### Conclusion and final remarks

In conclusion, the SHAT-PS was successfully contextualized and psychometrically validated for use in Nigerian primary schools. The tool demonstrated strong reliability and a coherent six-factor structure. These findings highlight its potential as a valuable instrument for assessing and informing school health practices in Nigeria. By quantifying cultural and institutional factors, the SHAT-PS provides a framework for identifying needs in areas such as health services, education, and infrastructure. However, while the results are promising, the current findings are based on data from a specific region and stakeholders. Broader validation across diverse geographic, socio-economic, and stakeholder groups is necessary before recommending the tool for national or large-scale implementation. Future research should build on this foundation to ensure the SHAT-PS’ broader applicability and long-term utility in guiding policy and practice.

*"The damage done in one year can sometimes take ten or twenty years to repair. An educated mind is the best tool for this restoration"*.

*– Chinua Achebe (The Trouble with Nigeria*,* 1983).*

## Data Availability

The datasets used and/or analyzed during the current study are available from the corresponding author, Danilo Garcia, upon reasonable request. Please contact danilo.garcia@icloud.com to obtain access to the raw data analyzed in this study.

## Abbreviations

CFI: Comparative Fit Index
SHAT-PS: School Health Assessment Tool for Primary Schools
SRMR: Standardized Root Mean Square Residual
RMSEA: Root Mean Square Error of Approximation
TLI: Tucker-Lewis Index
WHO: World Health Organization
